# Surgical management of choroid plexus papilloma of the cerebellopontine and cerebellomedullary angle: classification and strategy

**DOI:** 10.1007/s10143-021-01506-4

**Published:** 2021-02-24

**Authors:** S. D. Adib, J. M. Hempel, K. Kandilaris, F. Grimm, R. Evangelista Zamora, M. Tatagiba

**Affiliations:** 1 Department of Neurosurgery, University of Tuebingen, Hoppe-Seyler-Str. 3, 72076 Tuebingen, Germany; 2 Department of Neuroradiology, University of Tuebingen, Hoppe-Seyler-Str. 3, 72076 Tuebingen, Germany; 3 Department of Neuropathology, University of Tuebingen, Hoppe-Seyler-Str. 3, 72076 Tuebingen, Germany

**Keywords:** Choroid plexus papilloma, Cerebellopontine angle, CPA, Facial nerve, Bochdalek’s flower baskets, Ectopic choroid tissue

## Abstract

Choroid plexus papillomas (CPPs) are primary neuroectodermal neoplasms that usually arise in the fourth ventricle in adults. In this study, we present 12 patients with CPP arising from the cerebellopontine angle (CPP-CPA) and/or of the cerebellomedullary angle (CPP-CMA) that were treated in our department. Patients who underwent surgery for the treatment for CPP-CPA/CMA from January 2004 to March 2020 were identified by a computer search of their files from the Department of Neurosurgery, Tübingen. CPPs were classified according to their location into type 1 (tumor portion only in the CPA,), type 2 (tumor portions only in the CMA), and type 3 (tumor portions both in the CPA and CMA). Patients were evaluated for initial symptoms, previous therapies in other hospitals, extent of tumor resection, recurrence rate, and complications by reviewing patient documents. Of approximately 1500 CPA lesions, which were surgically treated in our department in the last 16 years, 12 patients (mean age 42 ± 19 years) were found to have CPP-CPA/CMA. Five were male, and seven were female patients. Gross total resection was achieved in nine cases, and a subtotal resection was attained in three cases. Tumor recurrence in the same location after the first surgery in our hospital was observed in 2 patients after 15 and 40 months of follow-up, and in another patient, distant metastases (C3/4 and L3 levels) were observed. Surgical removal of CPP is the treatment of choice, but additional therapeutic options may be necessary in case of remnant tumor portions, recurrence, or malignant transformation.

**Supplementary Information:**

The online version contains supplementary material available at 10.1007/s10143-021-01506-4.

## Introduction

Choroid plexus papillomas (CPPs) are rare, benign [39], macroscopic “red or grayish-pink friable” (with a cauliflower appearance) [32], slow-growing [42], primary neuroectodermal neoplasms [38] that usually arise in the fourth ventricle in adults [39], and in the lateral ventricle in children [39]. They account for 0.3–0.7% of all intracranial tumors [32, 38]. CPPs can be classified according to their location, namely, classical intraventricular, extraventricular and intraventricular, and primary extraventricular. Between 9 and 20.5% of CPPs are located in the cerebellopontine angle (CPA) [24, 35], and the lesion size usually ranges from 2.5 to 4.7 cm [24]. However, many authors did not separate CPP of the CPA from CPP of the cerebellomedullary angle (CMA) and sum up all these lesions as CPA lesions; although, these conditions have differences in terms of their relation to different cranial nerves and surgical strategy. Surgical removal of CPP is the treatment of choice [2, 24, 32], but additional therapeutic options may be necessary in case of remnant tumor portions, recurrence, or malignant transformation.

With the above background, this retrospective study aimed to analyze the surgical management of CPP-CPA and CPP-CMA, with a focus on the clinical presentation, previous treatments in other hospitals, surgical strategy, the extent of resection, and early and late treatment outcomes.

## Materials and methods

### Data collection and inclusion criteria

Patients who had undergone surgery for CPP-CPA and CPP-CMA from January 2004 to March 2020 were identified through a computer search of the patients’ medical files at our neurosurgery department. Twelve patients with CPP-CPA/CMA from among >1500 patients with CPA lesions had undergone surgery in our department. CPP had been confirmed histopathologically in all cases. Two patients had undergone a second surgery due to recurrence.

Patients were evaluated for initial symptoms, previous treatments in external hospitals, tumor resection strategy, extent of tumor resection, recurrence rate, and complications by reviewing surgical and pathological reports, patient documents, neuroradiological data, and follow-up data. Furthermore, the preoperative and postoperative cranial nerve functions were assessed.

The study was approved by the ethics committee of the University Hospital Tübingen, Germany (reference no. 280/2017BO2).

### Classification

**Fig. 1:**
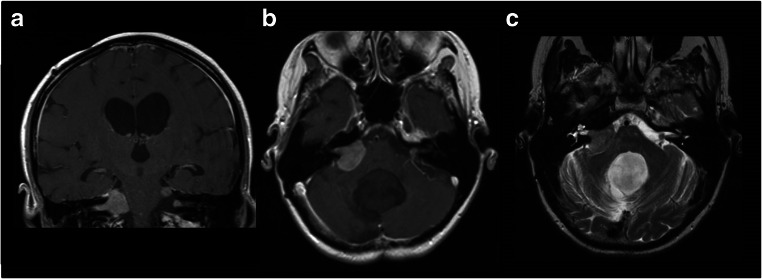
Coronal (**A**) and axial (**B** + **C**) MRI of type 1 choroid plexus papilloma: tumor portions are present in the cerebellopontine angle, without any tumor portions in the cerebellomedullary angle (superior to the line between the pons and medullar oblongata) (this patient had also drop metastases in the contralateral cerebellopontine angle)

**Fig. 2:**
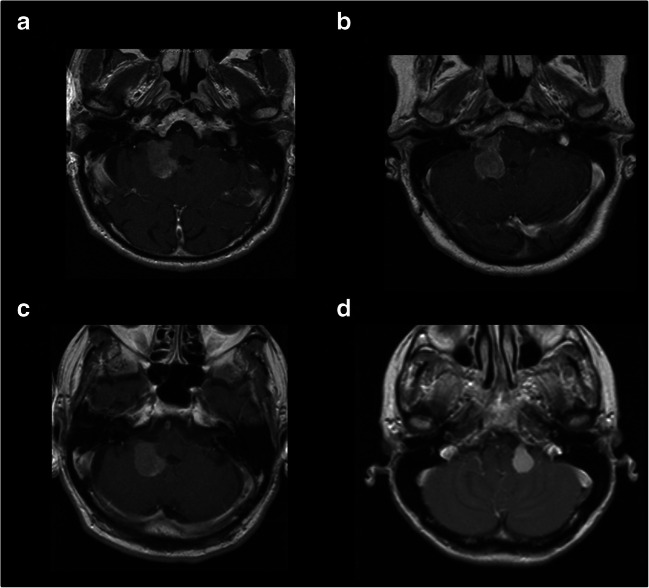
Axial MRI (**A**, **B**, **C, D**) of different Type 2 choroid plexus papillomas: tumor portions are present in the cerebellomedullary angle (inferior to the line between the pons and medullar oblongata), without any tumor portions in the cerebellopontine angle

**Fig. 3:**
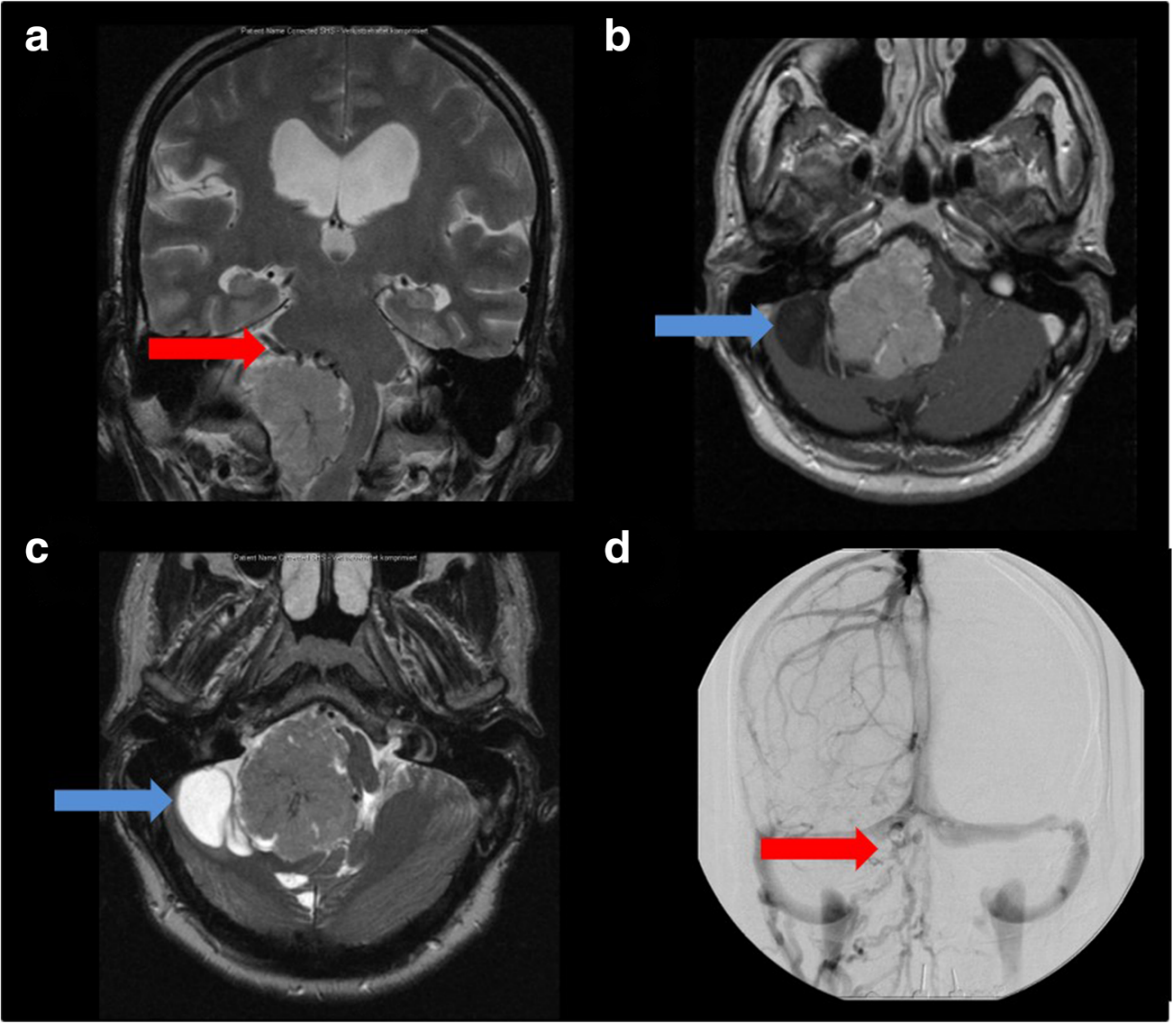
Coronal (**A**) and axial (**B** + **C**) MRI of type 3 choroid plexus papilloma: tumor portions are present in the cerebellopontine angle (CPA) and cerebellomedullary angle (CMA) (tumor portions superior and inferior to the line between the pons and medullar oblongata); angiography (**D**) revealed that the blood supply to the tumor arose from the right anterior inferior cerebellar artery and right posterior inferior cerebellar artery

CPPs with location lateral to the brainstem were classified according to the line between the pons and the medulla oblongata:

- Type 1: tumor portions in the CPA, without any tumor portions in the CMA (superior to the line between the pons and medullar oblongata) (Fig. 1)
- Type 2: tumor portion in the CMA, without any tumor portions in the CPA (inferior to the line between the pons and medullar oblongata) (Fig. 2)
- Type 3: tumor portion in the CPA and CMA (tumor portions superior and inferior to the line between the pons and medullar oblongata) (Fig. 3)

### Surgical technique: positioning, approach, and tumor resection strategy

All patients received general anesthesia and intraoperative monitoring according to our standards. Intraoperative monitoring included motor-evoked potentials (MEP) and sensory-evoked potentials (SEP) of the upper and lower extremity, furthermore MEP and electromyography (EMG) recordings of the facial nerve, auditory-evoked potentials (AEP). In case of type 2 and type 3 CPP, intraoperative monitoring of the lower cranial nerves was recorded. The anesthesiologic setup in the case of semi-sitting position included transesophageal echocardiography for early detection of air emboli.

Positioning and approach were dependent on the location of the main tumor portion. In patients in whom the main tumor portion is located in the CPA, the retrosigmoid approach in the semi-sitting position was performed, whereas the medial suboccipital-subtonsillary approach (according to Herlan et al. [17]) in the prone position was performed if the main tumor portion is located in the CMA. If further tumor portions are located in the level of the foramen magnum and upper cervical spine, an additional laminectomy of the C1 was performed.

The tumor resection strategy included (1) coagulation of the major feeding vessels, (2) debulking of the tumor with cavitron ultrasonic surgical aspirator (CUSA), (3) intraoperative rapid pathological analysis, (4) removal of the tumor, and (5) coagulation of any fragment of the tumor that may remain.

### Follow-up

Postoperative magnetic resonance imaging (MRI) was performed 3 months after surgery. One patient was not re-examined in our hospital because further oncological therapy was carried out in his home hospital. The most extended follow-up period of the 11 other patients ranged from 3 to 75 months (mean follow-up 26.2 months).

### Statistical analysis

Statistical analysis was performed in SPSS (IBM SPSS Statistics for Windows, Version 22.0. Armonk, NY: IBM Corp.). First, the continuous data were examined for normal distribution using the Kolmogorov-Smirnov test and the Shapiro-Wilk test. The non-normally distributed data were tested using the nonparametric Mann-Whitney *U* test to determine whether there were differences in the distribution of age among the WHO groups.

A chi-square test of homogeneity was performed to evaluate whether the proportions between the WHO groups and the nominal variables sex, tumor type, side, positioning, previous external therapies, the extent of resection, recurrence, and further surgeries differ.

The same test was carried out to estimate differences between the groups of three different tumor types with the above-mentioned variables.

On significant differences, a post hoc analysis involved pairwise comparisons using the *z*-test of two proportions with a Bonferroni correction.

## Results

Twelve patients (five were male and seven were female) with CPP-CPA/CMA were included in this study. The mean patient age was 42 ± 19 years. Seven CPP were located on the right side, and four were on the left side (Table 1). One patient had bilateral CPP (but only the right one was removed).

**Table 1:** Relation between type of the tumor, grade of the tumor, oncological therapy, the extent of resection, recurrence rate, follow-up and surgical management

	Age	Sex	Type	Side	WHO grade	Previous external therapies	Extent of resection	Recurrence	Further surgeries in our hospital	FU (months)	Positioning (1)	Approach (1)	Positioning (2)	Approach (2)
1	22	w	3	Left	WHO I	None	GTR	No	None	4	Prone position	Median suboccipital	x	x
2	32	w	2	Right	WHO I	None	GTR	No	None	3	Prone position	Median suboccipital	x	x
3	17	m	2	Left	WHO I	None	STR	No	None	18	Prone position	Medial suboccipital	x	x
4	51	m	2	Right	WHO II	Two previous surgeries + cyberknife	STR	x	x	x	Prone position	Medial suboccipital + laminectomy	x	x
5	47	w	1	Right + left	WHOII	3 surgeries + chemotherapy + radiotherapy	GTR	Yes	Removal of two spinal metastases	69	Semi-sitting position	Retrosigmoid	x	x
6	64	w	2	Left	WHO I	None	GTR	No	None	41	Prone position	Medial suboccipital + laminectomy	x	x
7	38	w	3	Left	WHO II	None	GTR	No	None	75	Prone position	Medial suboccipital	x	x
8	49	w	3	Right	WHO I	None	GTR	No	None	4	Semi-sitting position	retrosigmoid	x	x
9	11	m	2	Right	WHO I	None	GRT	No	None	5	Prone position	Medial subocipital	x	x
10	67	w	3	Right	WHO I	One previous surgery	STR	Yes	Second surgery	14	Semi-sitting position	Retrosigmoid	Prone positioning	Medial suboccipital
11	70	m	3	Right	WHO I	None	GTR	Yes	Second surgery	37	Semi-sitting position	Retrosigmoid	Prone positioning	Medial suboccipital
12	37	m	3	Right	WHO I	None	GTR	No	None	6	Semi-sitting position	Retrosigmoid	x	x

*FU*, follow-up; *GTR*, gross total resection; *STR*, subtotal resection

### Radiological findings

Of the 12 patients, in 11 patients preoperative and postoperative MRI data were available. In one case, only the postoperative MRI was available. Still, the patient’s surgical report described a CPP of the CPA and CMA, and postoperative computed tomography image revealed a retrosigmoid approach (also confirmed by surgical report).

One patient had type 1, five patients had type 2, and six cases had type 3 CPPs (Table 1). MRI revealed cystic CPP-CPA in one case (1/11), brainstem compression with displacement in six patients (6/11), and secondary hydrocephalus in four patients (4/11). The Evans index in the preoperative MRI of the 11 cases was between 0.25 and 0.40 (mean 0.31 ± 0.06). In two cases, the CPPs were multiloculated, and in two patients, type 3 CPP also had tumor portions in the foramen magnum and cervical spinal canal.

### Angiography

One patient underwent angiography before surgery (case 12, Fig. 3), which revealed the blood supply of the tumor originated from the right anterior inferior cerebellar artery (AICA) and the right posterior inferior cerebellar artery (PICA).

### Previous therapies

Before the first surgery in our department, one patient with multiple CPP metastases (case 5) had undergone 3 surgeries in other hospitals, chemotherapy (etoposide, carboplatin, and vincristine), and radiotherapy; one patient (case 4) underwent 2 surgeries in other hospitals and a cyberknife therapy; and one patient had undergone one surgery (case 10) (Table 1).

### Presenting symptoms

The most common symptoms were headache (7/12) and dizziness (7/12). Other symptoms included walking disturbance and ataxia (4/12), hearing impairment (4/12), a disorder of coordination and sensitivity of the arm (3/12), nausea (2/12) and vomiting (1/12), facial nerve palsy (1/12) on the side of the CPP-CPA (H + B IV), trigeminal hypesthesia (1/12), nystagmus (1/12), papilledema (1/12), dysphagia (1/12), and dysarthria (1/12).

In summary, nine of 12 patients (9/12) had symptoms associated with cranial nerve dysfunction (dizziness, hearing impairment, facial nerve palsy, trigeminal hypesthesia, nystagmus, dysphagia, and dysarthria). Considering our classification, a clear relation was found between cranial nerve dysfunction and tumor type.

### Histopathological results

CPP was histologically confirmed in each case (Table 1) with typical characteristics such as papillary architecture, membranous staining for Kir7.1; and no or low mitotic activity.

**Fig. 4:**
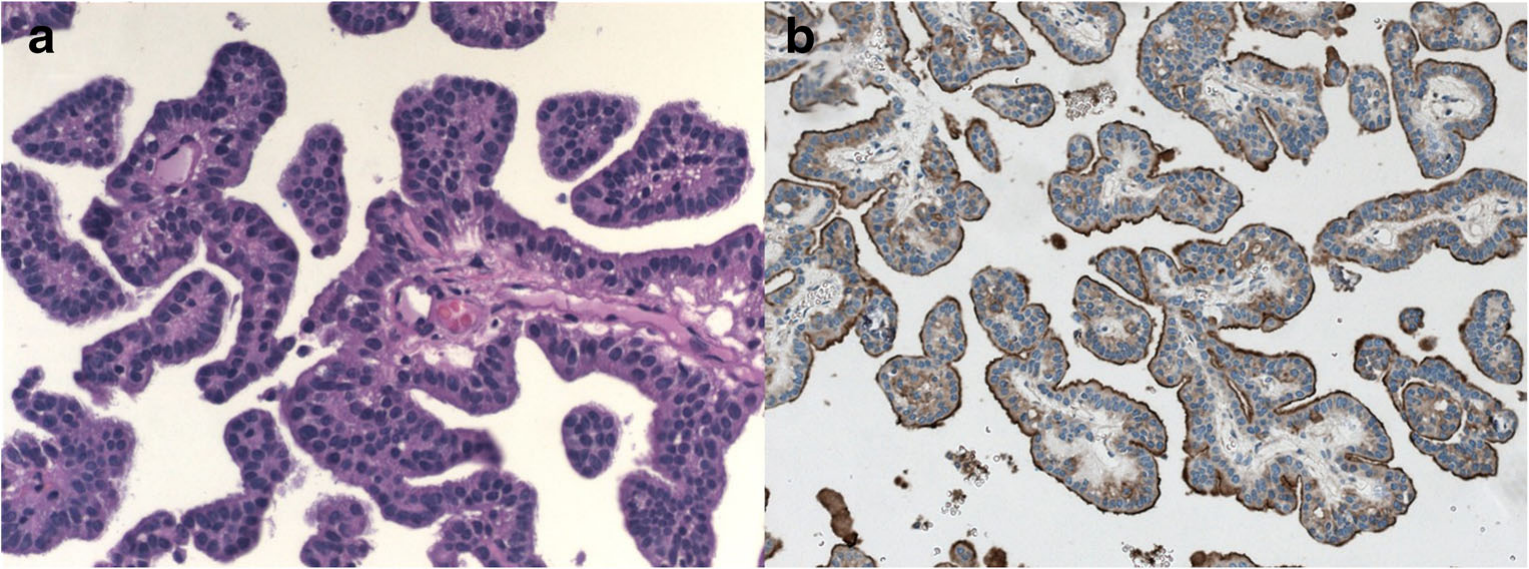
**A** Well-differentiated papillary pattern composed of a monolayer of monomorphic round cells (HE × 400); **B** clear membranous staining for Kir7.1 (× 200)

The histopathological analysis revealed CPP WHO grade I (Fig. 4 A + B) in nine patients who had undergone first surgery in our department and an atypical CPP (WHO grade II) in three patients (with two previous surgeries in an external hospital (cases: 4, 5, 7). Two of the patients had a second surgery in our department due to recurrence (cases: 10 and 11), and one of the cases had undergone further surgery due to metastases in the spinal canal (case 5); and in patient 10, the CPP became an atypical CPP (WHO II).

### Positioning and approach

Of the 12 patients, 5 received surgery in semi-sitting position through retrosigmoid approach, and 7 received surgery through a median suboccipital-subtonsillary approach in the prone position; and in two of the these patients (patients 4 and 6), an additional laminectomy of the C1 arc was performed because the tumor portions were located in the level of the foramen magnum and upper cervical spine (Table 1).

Two of the patients (cases 10 and 11), with first surgery in our department in the semi-sitting position with the retrosigmoid approach, received a second surgery in our department (second time in prone position through the medial suboccipital approach).

### CPP WHO I

The neuropathological examination after the first surgery in our department revealed a CPP (WHO I) in nine patients (Table 1). Seven of the patients with CPP WHO I was treated with only one surgery in our department (they had no previous or further therapy) because there were no recurrence and remnant tumors after surgery (patients 1, 2, 3, 6, 8, 9, and 12).

One of the patients with CPP WHO I had undergone first surgery in an external hospital 8 years ago (4. ventricle CPP) (case 10); the second surgery and one further surgery were performed in our department after 1 year due to transformation to an atypical CPP WHO (second surgery WHO II). Patient 11 had undergone a second surgery 3 years after the first surgery in our department, and no further recurrence was recorded after the second surgery.

### Atypical CPP WHO II

Patients 4, 5, and 7 had an atypical CPP in their neuropathological examination after the first surgery in our department. One of the patients with atypical CPP WHO II had no previous and further therapy, because there was no recurrence and remnant tumor after surgery (Table 1: patient 7). Patient 4 had two prior surgeries in another hospital and one previous cyberknife therapy. After surgery in our department, no further follow-up was done in our department (Table 1). Patient 5 had a metastasized atypical CPP. He had two previous surgeries in other centers and a previous chemotherapy with etoposide, carboplatin, and vincristine, and radiotherapy. After CPP-CPA surgery in our center and shunt therapy, he underwent two further surgeries for the metastases of the spine at L3 and C3/4 levels. Since multiple metastases were detected during follow-up, he received no additional therapies as palliative therapy, except for a percutaneous endoscopic gastrostomy.

### Extent of resection, recurrence, and further oncological therapies

**Fig. 5:**
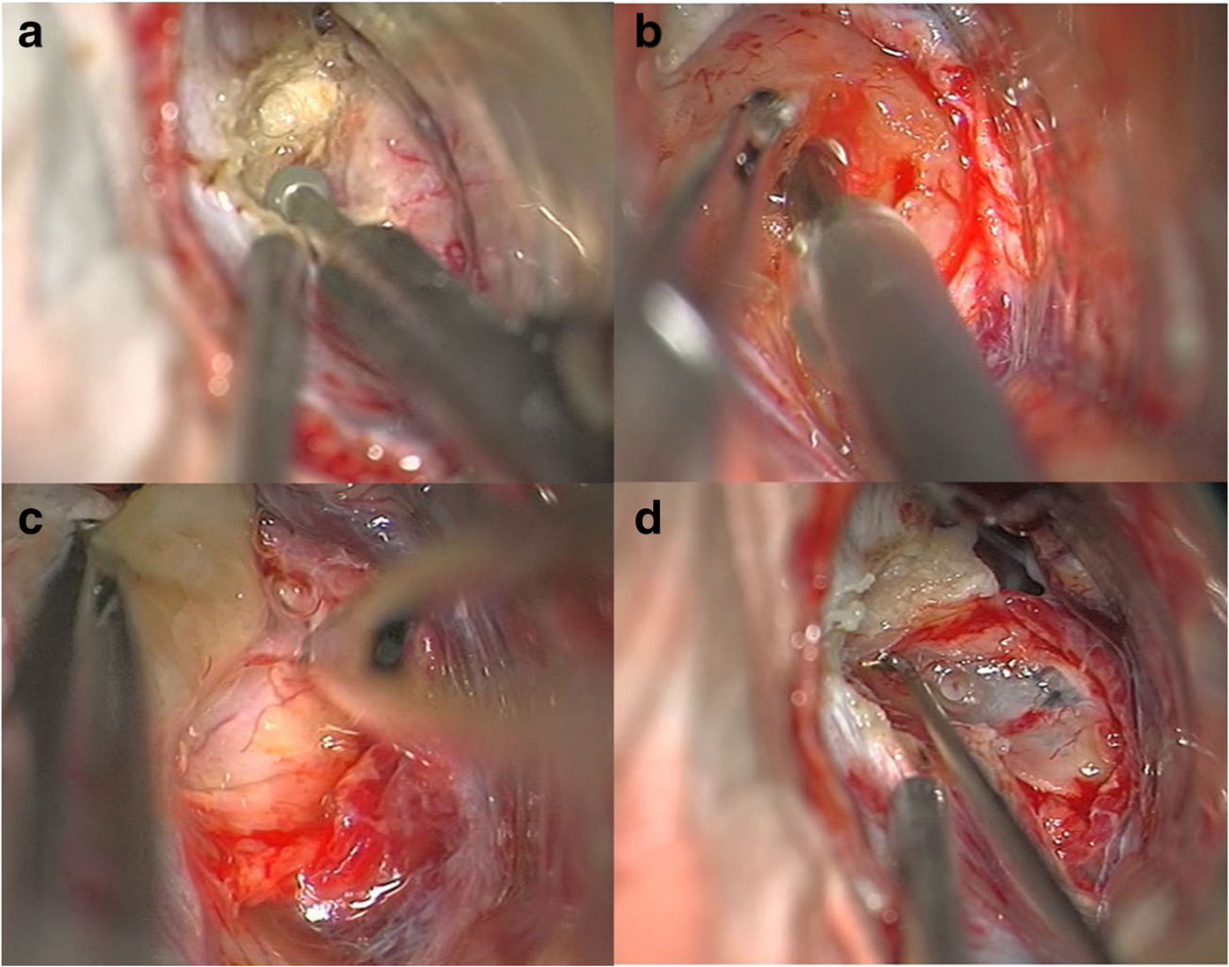
Removal of a type 1 choroid plexus papilloma: **A** opening of the internal auditory canal, **B** debulking of the tumor (using an ultrasonic aspirator), **C** the tumor is dissected from surrounding structures, **D** gross total resection was achieved

Gross total resection was achieved in nine cases, and subtotal resection was attained in three cases (Table 1, Fig. 5). Three patients already had tumor recurrence (after previous surgery/surgeries in external hospitals) during the first surgery in our hospital (Table 1). Two of them had received radiotherapy during the first presentation in our hospital (one had cyberknife therapy and one had fractionated radiotherapy) (Table 1: patient 4 and patient 5). One of the patients had already received chemotherapy (Table 1: patient 5).

### Postoperative cranial nerve function

Anatomical facial nerve preservation was achieved in all 12 patients, and in 11 patients, no worsening of facial nerve function was detectable. In one patient, the tumor had no contact to the lower cranial nerves (type I). In five of the 12 cases, the tumor involved only the CMA (type 2) without contact to cranial nerves VII and VIII. From the other seven cases with contact to cranial nerves VII and VIII (types 1 and 3) immediately after surgery, two patients experienced dizziness (2/12) and one patient had impaired facial nerve function (from H + B grade IV to H + B grade V).

As regards to the lower cranial nerve function (cranial nerves IX, X, XI, and XII) immediately after surgery, two patients developed dysphagia and one patient developed dysarthria. Seven patients had no new symptoms after surgery, except for the usual mild headache and nausea.

### Follow-up and tumor recurrence after surgery in our department

One patient (patient 4) had no follow-up after surgery in our department. The mean follow-up duration of the other patients was 27.2 months (Table 1). Tumor recurrence in the same location after the first surgery in our hospital was observed in two patients at 15 months and 40 months of follow-up (Table 1: cases 10 and 11), and in one further patient distant metastases (in the levels of C3/4 and L3) were observed (case 5). All three patients had undergone further surgery. The patient developed distant metastases after the second surgery, and palliative treatment was employed for multiple metastases.

### Complications

Further complications (besides cranial nerve dysfunction) immediately after surgery included two cases of persistent hydrocephalus requiring shunt implantation. No mortality occurred during the follow-up, but in one patient, the therapeutic concept was changed after further surgeries of metastases in the spinal canal during the following years, to palliative care (15 years after CPP-CPA surgery) (case 5).

### Statistical analysis

1. Mann-Whitney *U* test:
  The Mann-Whitney *U* test demonstrated that there was no significant difference between the group of patients with CPP WHO grade I and with CPP WHO grade II regarding their age (*p* = .727).
2. Differences between the groups of patients with different tumor types regarding sex, WHO grade, extent of resection, recurrence, positioning, previous external therapy, and further surgery in our department:

There was no significant difference between the groups of patients with different tumor types regarding sex (*p* = .454), WHO grade (*p* = .193), extent of resection (*p* = .561), and recurrence (*p* = .087).

There was a significant difference between the groups of different tumor types regarding positioning (*p* = .038), previous external therapy (*p* = .027), and further surgeries in our department (*p* = .007); The post hoc analysis revealed statistical differences between the types 1 and 2.

3) Differences between the patients with different WHO grade regarding sex, side of tumor, positioning, previous external therapies, extent of resection, recurrence, further surgeries in our department, and type of tumor:

There were no significant difference between the group of patients with CPP WHO grade I to the group of patients with CPP WHO grade II regarding sex (*p* = .735), side of tumor (*p* = .180), positioning of the patients (*p* = .735), previous external therapies (*p* = .064), extent of resection (*p* = .0700), recurrence (*p* = .700), further surgeries in our department (*p* = .157), and WHO grade (*p* = .193).

## Discussion

### Choroid plexus papilloma of the cerebellopontine angle: first series, location, and origin

CPP was first described by Guerard in 1833 [3, 6], and the first case of CPP-CPA was reported in Cushing’s monograph on tumors of the acoustic nerve [38]. Since then, further cases were reported in the literature [1, 5, 8, 12, 19–21, 33, 36]. Tasdemiroglu et al. [42] concluded that CPP-CPA usually develops between the second and fifth decades of life; in contrast, Posey [34] reported that 50% of the cases occurred before age 20 years [15, 30, 37] and congenital cases have been reported.

The CPA is the most primary extraventricular location of CPP [2]; however, many authors do not separate CPP of the CPA and CPP of the CMA and classified all these lesions as CPA lesions. From the surgical perspective, there is a considerable difference between these conditions because CPP-CPA and CPP-CMA are located close to different cranial nerves (cranial nerves VII and VIII versus lower cranial nerves) and different positioning, approaches, and strategies are necessary. The origin of CPP-CPA is still under debate [24], since CPP-CMA [28] usually arises from the choroid plexus of the foramen of Luschka.

There are different hypotheses regarding the origin of CPP-CPA: (1) Tasdemiroglu et al. [42] assumed that CPP-CPA developed from embryonic choroidal remnants. (2) Greene et al. [14] supposed (in 1951) that CPPs of nonventricular origin arise from the ectopic choroid tissue (so-called Bochdalek’s flower baskets) [32, 38, 42] and separate them into primary and secondary ectopia. Primitive ectopic secretory choroid plexuses within the brain substance are considered primary ectopia, and the segregation of choroidal tissue during the developmental stage of the brain is regarded as secondary ectopia. (3) CPP-CPA may develop as a direct extension of the primary intraventricular papilloma through the foramen of Luschka (type 3 of our classification). (4) Luo et al. [24] presumed that CPP-CPA develops from drop metastases or seeding along with the cerebrospinal fluid (CSF) pathways from an intraventricular CPP [10, 16, 24] (patient 5).

### Biological behavior: WHO classification and malignant transformation

The WHO classified choroid plexus tumors into CPP (WHO I), atypical CPP (WHO II), and choroid plexus carcinoma (WHO III) [22, 24]. Atypical CPP (WHO grade II) shows increased mitotic activity (≥2 mitoses per 10 high-power fields) with features intermediate between CPP and choroid plexus carcinoma. Naguib et al. [30] concluded that the determination of malignancy in CPP is also based on marked invasion of adjacent neural tissues (or even invasion of mastoid air cells) and a loss of the regular papillary pattern with the “presence of malignant features of the cells.”

In our series, 75% of the patients (9/12) had a CPP (WHO I) and 15% (3/12) had an atypical CPP (WHO II) in the first neuropathological analysis in our department. Panizza et al. [32] mentioned that 10% of CPP cases are malignant and that between 10 and 30% of CPP cases show malignant transformation during the disease course [32, 40, 41]. In our series, one patient (case 10) (8.33%) showed a transformation from a CPP (WHO I) to an atypical CPP (WHO II). Tanaka et al. [40] concluded that the mechanism of atypical transformation and rapid regrowth in CPP is not entirely understood, but that vascular alterations might play a role in the impairment of the tumor microcirculation and that several growth factors are involved, such as insulin-like growth factor II and vascular endothelial growth factor. Coincidental CPP-CPA together with vestibular schwannoma [23] and meningioma [27] has been reported.

### Symptoms

In our study, the most common symptoms were associated with cranial nerve dysfunction (9/12), and the second most symptoms were headache (7/12) and dizziness (7/12). These findings support the findings of Luo et al. [24] who concluded that the most frequent symptom of CPP-CPA is headache (in some cases due to hydrocephalus) and of Tasdemiroglu et al. [42] who summarized that the most common symptoms of CPP-CPA are dysfunction of cranial nerves V, VII, and VIII and ataxia [18, 42]. Simonati et al. [36] concluded that early signs were even symptoms of elevated intracranial pressure and hearing impairment.

### Radiological findings: MRI, CT, multiple cysts, calcification, bone erosion, hydrocephalus

Martin et al. [25] concluded that CPP appears homogenous on MRI, with low intensity or iso intensity on T1-weighted sequences. Still, they sometimes show “foci of high-signal intensity due to intratumoral hemorrhage” and flow voids due to high-flow feeding vessels. Usually, they offer a gadolinium enhancement.

Panizza et al. [32] concluded that in 3.7% of cases, multiple papillomas involving more than one site have been noted. In our study, multiple CPPs were observed in patients 5 and 10. Moreover, 4.1–20% of CPP cases show usual calcification in CT images [4, 11, 26, 32, 35]; on the other hand, they demonstrate erosion of surrounding bone in some cases [32]. Furthermore, 20% of CPP cases have cystic components [24]. In our series, one case had cystic components (8.33%) (patient 12, Fig. 3). Tanaka et al. [40] reported that cyst development might be associated with overproduction of CSF by the tumor or spontaneous bleeding into the tumor. Cystic CPP may appear as purely cystic tumors or cystic tumors with multiple mural nodules [9, 29]. Tomita et al. [43] summarized that some cases might show contrast enhancement in the cyst wall due to inflammatory reaction after hemorrhage.

### Angiography

In our series, patient 12 underwent angiography before surgery to detect the main supplying blood vessels, because of huge tumor vessels in the MRI (Fig. 3), which revealed that the blood supply to the tumor arose from the right AICA and right PICA.

Since CPP are vascular tumors, digital subtraction angiography may help analyze the blood supply and may reveal “blushes” (CPP-characteristic enlarged/hypertrophic arterial feeders), “early filling of veins draining from the tumor” [25, 32], or intratumoral arteriovenous shunting, mimicking a hemangioblastoma in this location [4, 13, 25, 32]. On the contrary, in our opinion, angiography is not necessary for every CPP-CPA or CPP-CMA.

The usual blood supply to the choroid tuft of the CPP-CPA arises from the AICA [46, 47], like in our case (in some cases, from the middle cerebellar artery or branches of the external carotid artery or even from the external carotid artery, which may cause some confusion, since it is the usual blood supply source in meningiomas of the CPA). A pathognomonic characteristic of CPP is an enlargement of this artery with dilated branches (Fig. 1, red arrows).

### Hydrocephalus and shunt therapy

Six patients (6/11) demonstrated brainstem compression, and in four patients (4/11), hydrocephalus was observed. The Evans index from the 11 patients with preoperative MRI ranged from 0.25 and 0.40 (mean 0.31 ± 0.06). In two patients (2/12), shunt therapy was necessary 1 and 2 months after surgery. Martin et al. [25] concluded that hydrocephalus is often associated with CPP. CSF hypersecretion by the tumor and occlusion of CSF pathways (fourth ventricle obstruction) are the main mechanisms behind hydrocephalus [25]. In both cases with shunt therapy, an endoscopic transventricular third ventriculostomy was not indicated, because there were no obtructions of the venticular system diagnosted.

### Surgical strategy

Despite their proximity to each other, it is important to differentiate the CPA and CMA, because both locations are associated with different challenges. CPP might only involve the CPA, CMA, or both. Patient positioning, surgical approach, and strategy might be different as well.

For the surgical management of CPP-CPA, it is essential to realize whether the main portion of the tumor is located in the CPA or CMA (which is more common, because it arises from the choroid plexus of the fourth ventricle or the foramen of Luschka) and whether the tumor is extended to the foramen magnum.

We recommend coagulation of the feeding vessels before targeting the tumor itself. Luo et al. [24] and Panizza et al. [32] also suggested isolation and closure of the major feeding artery before debulking surgery. Tomita et al. [43] recommended intraoperative rapid pathological examination of different samples from the cyst wall to determine whether the cyst walls contain neoplastic cells. Luo et al. [24] mentioned that CUSA was sometimes used for total resection of the calcified tumor. Luo et al. [24] concluded that surgeons should try to remove as much of the tumor as possible, and Panizza et al. [32] recommended coagulation of any fragment of the tumor that may remain and that surgical treatment alone is, in most cases of WHO grade I CPP, adequate to prevent a recurrence. In case of strong adherence to the facial nerve, a subtotal removal should be performed because of the benign characteristics [32]. Panizza et al. [32] concluded that “dissemination of tumor cells along the CSF pathways, so-called seeding” is possible, which may occur in benign and malignant CPP.

### Extent of resection, survival rate, and recurrence

Luo et al. [24] summarized that gross total resection provided “durable tumor control” and was associated with a significant increase in overall survival (OS) and progression-free survival (PFS). Anderson et al. [2] conclude that the 5-year survival rate is 100% with gross total resection and 94% for subtotal resection of CPP. Luo et al. [24] reviewed that 5-year OS ranged from 81 to 100% and recurrence would occur in 6–25% of CPP cases.

### Other therapeutic options

CPP usually have intensive vascular supply; therefore, preoperative endovascular embolization may be discussed in some cases [24], especially in CPP with supratentorial intraventricular location [31, 44, 45]. Moreover, Di Rocco et al. [7] discussed preoperative chemotherapy with cisplatin to reduce vascular supply and tumor size. Naguib et al. [30] also tried preoperative radiation therapy. In agreement with Luo et al. [24], we think that there is no evidence for an increase in PFS for postoperative adjuvant therapy compared with surgery alone in case of CPP WHO I, and we agree that the “watch-and-wait” strategy following radical surgical resection would be the best choice. In case of incomplete resection [32] or recurrence or malignant transformation [40], radiotherapy should be explored [2]. Tanaka et al. [40] concluded that surgical specimens of CPP should be evaluated to identify mitotic figures and radiation therapy can be considered an adjuvant treatment. In our series, two patients received radiotherapy (patients 4 and 5) and one patient chemotherapy (patient 5).

### Limitations

The main limitation of this study is the retrospective design and the small number of patients, due to the rare prevalence of CPP-CPA/CMA. Furthermore, our series is very heterogeneous, as patients with recurrence, malignant transformation, metastases, and different WHO grades were analyzed.

## Conclusion

The pathophysiology of CPP-CPA/CMA is very complex due to the different WHO grades, malignant transformations, recurrence, various extensions, and origins; therefore, our series is very heterogeneous. We recommend separating CPP lateral to the brainstem into three groups according to their extension to the CPA and CMA. The most usual group is type 3, since tumors may arise from the choroid plexus of the foramen of Luschka. The management of CPP-CPA is complex, and different strategies are necessary to perform safe and successful surgery. Although surgery is the main therapy, other procedures such as preoperative embolization, radiotherapy, and chemotherapy should be explored. Radiotherapy should be performed in case of remnant tumor portions, recurrence, metastases, or malignant tumors.

## Supplementary Information

ESM 1 (RTF 458 kb)

## Data Availability

All data are included in the manuscript.
