# Supplementary material for: Surgical management of choroid plexus papilloma of the cerebellopontine and cerebellomedullary angle: classification and strategy
Source: Neurosurg Rev. 2021 Feb 24;44(6):3387–97. doi: 10.1007/s10143-021-01506-4 (PMC8592964; doi:10.1007/s10143-021-01506-4)
Supplement: Supplementary file 1 — (RTF 458 kb) [file 10143_2021_1506_MOESM1_ESM.rtf]

Supplementary Material: 


1. Mann-Withney-U test:
The non-normally distributed data were tested using the nonparametric Mann-Whitney-U test to determine whether there were differences in the distribution of age among the WHO-groups.


2. Differences between the groups of patients with different tumor types regarding sex, WHO grade, extent of resection, recurrence, positioning, previous external therapy and further surgery in our department:

2.1: A chi-square test of homogeneity was performed to evaluate whether the proportions between the tumor type and sex differ: 
Chi-Square Tests	
	Value	df	Asymptotic Significance (2-sided)	Exact Sig. (2-sided)	
Pearson Chi-Square	1.577a	2	.454	.747	
Likelihood Ratio	1.932	2	.381	.747	
Fisher's Exact Test	1.565			.747	
N of Valid Cases	12				

a. 6 cells (100.0%) have expected count less than 5. The minimum expected count is .42.	


2.2: A chi-square test of homogeneity was performed to evaluate whether the proportions between the tumor type and WHO grade differ: 

Chi-Square Tests	
	Value	df	Asymptotic Significance (2-sided)	Exact Sig. (2-sided)	
Pearson Chi-Square	3.289a	2	.193	.386	
Likelihood Ratio	3.085	2	.214	.386	
Fisher's Exact Test	2.741			.386	
N of Valid Cases	12				
a. 6 cells (100.0%) have expected count less than 5. The minimum expected count is .25.	

2.3: A chi-square test of homogeneity was performed to evaluate whether the proportions between the tumor type and positioning differ: 
Chi-Square Tests	
	Value	df	Asymptotic Significance (2-sided)	Exact Sig. (2-sided)	
Pearson Chi-Square	6.514a	2	.038	.034	
Likelihood Ratio	8.662	2	.013	.034	
Fisher's Exact Test	6.170			.034	
N of Valid Cases	12				
a. 6 cells (100.0%) have expected count less than 5. The minimum expected count is .42.	

2.4: A chi-square test of homogeneity was performed to evaluate whether the proportions between the tumor type and previous external therapies differ: 

Chi-Square Tests	
	Value	df	Asymptotic Significance (2-sided)	Exact Sig. (2-sided)	
Pearson Chi-Square	14.222a	6	.027	.250	
Likelihood Ratio	9.677	6	.139	.250	
Fisher's Exact Test	9.277			.250	
N of Valid Cases	12				

a. 12 cells (100.0%) have expected count less than 5. The minimum expected count is .08.	

2.5: A chi-square test of homogeneity was performed to evaluate whether the proportions between the tumor type and extent of resection differ: 
Chi-Square Tests	
	Value	df	Asymptotic Significance (2-sided)	Exact Sig. (2-sided)	
Pearson Chi-Square	1.156a	2	.561	.659	
Likelihood Ratio	1.359	2	.507	.659	
Fisher's Exact Test	1.354			.659	
N of Valid Cases	12				

a. 6 cells (100.0%) have expected count less than 5. The minimum expected count is .25.	

2.6: A chi-square test of homogeneity was performed to evaluate whether the proportions between the tumor type and recurrence differ: 
Chi-Square Tests	
	Value	df	Asymptotic Significance (2-sided)	Exact Sig. (2-sided)	
Pearson Chi-Square	4.889a	2	.087	.159	
Likelihood Ratio	5.858	2	.053	.159	
Fisher's Exact Test	4.127			.159	
N of Valid Cases	12				

a. 6 cells (100.0%) have expected count less than 5. The minimum expected count is .25.	

2.7: A chi-square test of homogeneity was performed to evaluate whether the proportions between the tumor type and further surgeries in our department differ: 

Chi-Square Tests	
	Value	df	Asymptotic Significance (2-sided)	Exact Sig. (2-sided)	
Pearson Chi-Square	14.222a	4	.007	.038	
Likelihood Ratio	9.677	4	.046	.038	
Fisher's Exact Test	7.513			.038	
N of Valid Cases	12				


3.Differences between the patients with different WHO grade regarding sex, side of tumor, positioning, previous external therapies, extent of resection, recurrence, further surgeries in our department and type of tumor: 

3.1: A chi-square test of homogeneity was performed to evaluate whether the proportions between the WHO grade and sex differ: 
Chi-Square Tests	
	Value	df	Asymptotic Significance (2-sided)	Exact Sig. (2-sided)	Exact Sig. (1-sided)	
Pearson Chi-Square	.114a	1	.735	1.000	.636	
Continuity Correctionb	.000	1	1.000			
Likelihood Ratio	.116	1	.733	1.000	.636	
Fisher's Exact Test				1.000	.636	
N of Valid Cases	12					

3.2: A chi-square test of homogeneity was performed to evaluate whether the proportions between the WHO grade and side differ: 
Chi-Square Tests	
	Value	df	Asymptotic Significance (2-sided)	Exact Sig. (2-sided)	
Pearson Chi-Square	3.429a	2	.180	.268	
Likelihood Ratio	3.256	2	.196	.427	
Fisher's Exact Test	2.948			.268	
N of Valid Cases	12				

	

3.3: A chi-square test of homogeneity was performed to evaluate whether the proportions between the WHO grade and positioning differ: 
Chi-Square Tests	
	Value	df	Asymptotic Significance (2-sided)	Exact Sig. (2-sided)	Exact Sig. (1-sided)	
Pearson Chi-Square	.114a	1	.735	1.000	.636	
Continuity Correctionb	.000	1	1.000			
Likelihood Ratio	.116	1	.733	1.000	.636	
Fisher's Exact Test				1.000	.636	
N of Valid Cases	12					
a. 3 cells (75.0%) have expected count less than 5. The minimum expected count is 1.25.	
3.4: A chi-square test of homogeneity was performed to evaluate whether the proportions between the WHO grade and previous external therapies differ: 
	
Chi-Square Tests	
	Value	df	Asymptotic Significance (2-sided)	Exact Sig. (2-sided)	
Pearson Chi-Square	7.259a	3	.064	.127	
Likelihood Ratio	7.217	3	.065	.127	
Fisher's Exact Test	6.189			.127	
N of Valid Cases	12				

a. 7 cells (87.5%) have expected count less than 5. The minimum expected count is .25.	

3.5: A chi-square test of homogeneity was performed to evaluate whether the proportions between the WHO grade and extent of resection differ: 

Chi-Square Tests	
	Value	df	Asymptotic Significance (2-sided)	Exact Sig. (2-sided)	Exact Sig. (1-sided)	
Pearson Chi-Square	.148a	1	.700	1.000	.618	
Continuity Correctionb	.000	1	1.000			
Likelihood Ratio	.142	1	.706	1.000	.618	
Fisher's Exact Test				1.000	.618	
N of Valid Cases	12					

a. 3 cells (75.0%) have expected count less than 5. The minimum expected count is .75.	
	

3.6: A chi-square test of homogeneity was performed to evaluate whether the proportions between the WHO grade and recurrence differ: 
Chi-Square Tests	
	Value	df	Asymptotic Significance (2-sided)	Exact Sig. (2-sided)	Exact Sig. (1-sided)	
Pearson Chi-Square	.148a	1	.700	1.000	.618	
Continuity Correctionb	.000	1	1.000			
Likelihood Ratio	.142	1	.706	1.000	.618	
Fisher's Exact Test				1.000	.618	
N of Valid Cases	12					
3.7: A chi-square test of homogeneity was performed to evaluate whether the proportions between the WHO grade and further surgeries in our hospital differ: 

Chi-Square Tests	
	Value	df	Asymptotic Significance (2-sided)	Exact Sig. (2-sided)	
Pearson Chi-Square	3.704a	2	.157	.291	
Likelihood Ratio	3.961	2	.138	.291	
Fisher's Exact Test	2.887			.291	
N of Valid Cases	12				

a. 5 cells (83.3%) have expected count less than 5. The minimum expected count is .25.	
